# The influence of maternal glucocorticoids on offspring phenotype in high- and low-risk environments

**DOI:** 10.1093/beheco/arab099

**Published:** 2021-09-10

**Authors:** Kirsty J MacLeod, Tracy Langkilde, Cameron P Venable, David C Ensminger, Michael J Sheriff

**Affiliations:** 1 Department of Ecosystem Science and Management, Pennsylvania State University, Forest Resources Building, University Park, PA, 16802, USA; 2 Department of Biology, Pennsylvania State University, Mueller Laboratory , University Park, PA, 16802, USA; 3 Department of Biology, Lund University, Sölvegatan 37, 223 62, Lund, Sweden; 4 Intercollege Graduate Degree Program in Ecology, The Huck Institute of the Life Sciences, Pennsylvania State University, University Park, PA, 16802, USA; 5 Center for Brain, Behavior and Cognition, Pennsylvania State University, University Park, PA, 16802, USA; 6 Department of Biological Sciences, San Jose State University, One Washington Square, San José, CA, 95192, USA; 7 Biology Department, University of Massachusetts Dartmouth, Dartmouth, MA, 02747, USA

**Keywords:** invasive predator, maternal effects, predation risk, *Sceloporus undulatus*, transgenerational phenotypic plasticity

## Abstract

Elevated maternal glucocorticoid levels during gestation can lead to phenotypic changes in offspring via maternal effects. Although such effects have traditionally been considered maladaptive, maternally derived glucocorticoids may adaptively prepare offspring for their future environment depending upon the correlation between maternal and offspring environments. Nevertheless, relatively few studies test the effects of prenatal glucocorticoid exposure across multiple environments. We tested the potential for ecologically relevant increases in maternal glucocorticoids in the eastern fence lizard (*Sceloporus undulatus*) to induce adaptive phenotypic changes in offspring exposed to high or low densities of an invasive fire ant predator. Maternal treatment had limited effects on offspring morphology and behavior at hatching, but by 10 days of age, we found maternal treatment interacted with offspring environment to alter anti-predator behaviors. We did not detect differences in early-life survival based on maternal treatment or offspring environment. Opposing selection on anti-predator behaviors from historic and novel invasive predators may confound the potential of maternal glucocorticoids to adaptively influence offspring behavior. Our test of the phenotypic outcomes of transgenerational glucocorticoid effects across risk environments provides important insight into the context-specific nature of this phenomenon and the importance of understanding both current and historic evolutionary pressures.

## Teaser

Offspring are affected by their mother’s experiences during gestation—but also by their own early life environment. We altered the levels of hormones associated with stress in female fence lizards, and found that the influence on offspring behaviours differed depending on how risky their environment was in early life. Offspring from “stressed” mothers in high-predation environments were harder to find, basked less, and stayed closer to the ground than those in low-predation environments.

## INTRODUCTION

Organisms frequently encounter ecological “stressors” within their environment, such as predation risk, conspecific aggression, and temperature extremes (Boonstra 2013). Such encounters result in activation of the hypothalamic-pituitary-adrenal (HPA) axis and subsequent release of glucocorticoid hormones, which facilitate organisms’ ability to cope with and react to such encounters (Wingfield et al. 1998; Sapolsky 2002). Although assumed to be an adaptive mechanism by which immediate survival is prioritized, prolonged exposure to elevated glucocorticoids can have detrimental effects on individuals through changes in metabolism, body condition, immune function, and behavior (De Vos et al. 1995; McCormick et al. 2014; Klein 2015; Osborne 2015), as well as reductions in survival and reproductive output (Sheriff et al. 2009; Zanette et al. 2011; MacLeod et al. 2018a, 2018b).

Elevated maternal glucocorticoid levels during gestation can also lead to phenotypic changes in offspring via transgenerational maternal effects (reviewed in Meaney et al. 2007; Love et al. 2013). Maternal effects—the causal influence of the maternal environment or phenotype on the offspring phenotype (Badyaev and Uller 2009; Mousseau et al. 2009)—are considered to be potentially important drivers of ecological and evolutionary dynamics, for example, by generating phenotypic variance that facilitates adaptive adjustment of offspring phenotype to local conditions (Räsänen and Kruuk 2007; Uller 2008). The phenotypic effects of maternally-derived glucocorticoids on offspring have largely been viewed as unavoidable negative outcomes of the adaptive physiological response to stressors of which glucocorticoid elevation is just one part (Seckl 2004; Seckl and Meaney 2004; Weinstock 2005). However, an alternative hypothesis is that such maternal effects may be “anticipatory” (Marshall and Uller 2007) such that maternally derived glucocorticoids act as “ecological integrators”, adaptively preparing offspring for the environment they are likely to experience, based on cues from the maternal environment (Dantzer et al. 2013; Sheriff and Love 2013; M. J. Sheriff et al. 2018). For example, high anxiety-like behavior was originally viewed as a negative outcome of prenatal stress (Brunton 2013; St-Cyr and McGowan 2015), but may increase survival in a high-stressor (e.g. predator-rich) environment by altering antipredator behaviors such as responsiveness to predator cues (Perrot-Minnot et al. 2017). Thus, glucocorticoid-driven maternal effects are likely to be context-dependent: adaptive when maternal and offspring environments are correlated, and maladaptive when the maternal and offspring environments are mismatched (Sheriff and Love 2013). Understanding this context-dependence is important in allowing us to better understand the importance of maternal effects generally, and glucocorticoid-driven maternal effects specifically, in ecological processes. Importantly, the efficacy of ecological integrators in linking maternal and offspring environments may depend on the stability of the environment over evolutionary time—for example, the introduction of novel predators may render historically adapted antipredator behaviors less effective (Donelan et al. 2020).

Although there is increasing appreciation that the effects of maternally-derived glucocorticoids on offspring traits are likely to be context-dependent (Sheriff and Love 2013; Sheriff et al. 2017), few studies have tested whether and how an offspring’s early life environment can modulate their phenotypic response to maternal glucocorticoids. Here, we tested: a) whether elevated maternal glucocorticoids resulted in changes in offspring phenotype in the eastern fence lizard *(Sceloporus undulatus)*; and b), whether phenotypic changes in offspring varied in the context of high- and low-predation risk from invasive fire ants (*Solenopsis invicta*), a key novel predator of fence lizards during early life (Darracq et al. 2017; Gifford et al. 2017). Despite their small size, fire ants are voracious predators capable of subduing relatively large animals by aggressively swarming and simultaneously stinging prey individuals (Langkilde 2009b). A study comparing juvenile fence lizard survival on islands of varying levels of fire ant density showed that lizard survival was halved in the high ant-density population relative to the low ant-density population (Gifford et al. 2017), demonstrating fence lizard vulnerability to this invasive predator.

The recent evolutionary history of eastern fence lizards with the invasive fire ant provides a useful system in which to test glucocorticoids as ecological integrators in the context of a novel predator (Donelan et al. 2020). Previous work has demonstrated that elevated glucocorticoid levels can induce behaviors that may have adaptive benefits in the context of fire ants, such as escaping to higher elevations in response to stressors (Trompeter and Langkilde 2011a). However, this may be a maladaptive response that increases predation from other sources, for example, aerial predators, which also contribute to predation of juvenile fence lizards (Thawley and Langkilde 2017). Crucially, potential benefits or costs of maternal glucocorticoid-induced phenotypes in the face of predation risk has not yet been tested. We predicted that compared to offspring from control mothers, those born to mothers with experimentally elevated glucocorticoid levels would be smaller, would show increased generalized anti-predator behavior (e.g. reduced latency to move and a higher likelihood of taking refuge in response to a handling stressor, and reduced activity), and would show greater subsequent growth and early-life survival in high-fire ant environments.

## METHODS

### Sceloporus undulatus capture and husbandry

Female *S. undulatus* (*N* = 44) were captured in April/May of 2017 from populations in southern Alabama. Upon capture, lizards were measured (mass to nearest 0.01 g, snout-vent length [SVL] to nearest 0.5 mm) and gravidity was assessed by abdominal palpation (Graham et al. 2012); all non-gravid females were released. Gravid females were transported back to facilities at the Pennsylvania State University, where they were individually housed in plastic containers (46 x 40 x 30 cm) furnished with a shelter and water bowl in a temperature-controlled room (21 ± 1˚C) until laying. A temperature gradient was maintained using a 60-W incandescent light bulb suspended over one end of each container, turned on for 8 hours a day (resulting in temperatures of ~32˚C at the hotter end and ~21˚C at the cooler end). Overhead lights were maintained on a 12:12 light:dark schedule to approximate natural conditions. When females were close to laying, we provided moist sand in which to lay eggs. Lizards were fed live food on alternate days (*Acheta domestica,* dusted with a calcium supplement [ReptiVite^TM^, Zoo Med Laboratories, Sacramento] twice weekly) and water was available *ad libitum*.

### Experimental glucocorticoid treatment

Gravid females were randomly assigned to a control or glucocorticoid treatment. From capture until laying, glucocorticoid treatment females (*N* = 20) received a thrice-weekly transdermal application of a corticosterone solution (hereafter CORT, the primary glucocorticoid in reptiles; (Meylan and Clobert 2005); 4 mg CORT [≥92%, Sigma C2505, Saint Louis, MO]/mL sesame seed oil vehicle). CORT doses were corrected for lizard body weight (0.2 μL/g lizard), resulting in doses of approximately 0.8 μg CORT/g body mass. Females in the control treatment (*N* = 24) received a dose of the sesame seed oil vehicle only. CORT or control solutions were applied to the center of the dorsal region by pipette between 07:30 PM and 08:30 PM without the need for handling. A CORT dose at this level has been shown to result in a short-term increase, with CORT levels returning to baseline 90 minutes post-dosing. In brief, a time-series experiment of 18 adult female lizards measuring CORT levels at 30, 60, 90, or 360 minutes post-dosing (with a further 8 lizards acting as controls to measure baseline levels of circulating CORT) showed that this dosage results in a short-term doubling of circulating CORT levels (from 9.97 ng/ml ± 1.81 to 21.77 ng/ml ± 4.37 at 30 mins post-dose) with a return to baseline by 90 minutes post-dose (further detailed in MacLeod et al. 2018b). This protocol mimics the magnitude of plasma CORT increases following a natural, non-lethal encounter with fire ants (McCormick et al. 2017). There is no evidence of strong diel patterns in CORT secretion in this species (Trompeter and Langkilde 2011b). Throughout treatment, housing containers were checked multiple times daily (without disturbing lizards) for signs of egg-laying. After laying, eggs were transferred to plastic containers (500 mL) filled with moist vermiculite (–200 kpa) and sealed to retain moisture, and transferred to incubators (30 ± 1^o^C) until hatching. Females that laid eggs before receiving a minimum of 3 doses or laid after receiving a maximum of 21 doses (6 weeks of treatment), were excluded from further experiments (*N* = 12). Of the remaining 32 females, 15 CORT-treated mothers produce a total of 56 offspring, and 17 control mothers produced a total of 65 offspring.

### Early life environment manipulation

We constructed two “high-fire ant predation risk” and two “low-fire ant predation risk” (treatment randomly assigned) open-air enclosures at the Solon Dixon Forestry Education Center (Andalusia, Alabama) by manipulating the density of an important juvenile lizard predator, invasive red fire ants (*Solenopsis invicta*). Each enclosure was 20 x 20 m, walled with aluminum flashing dug to a depth of ~30 cm and standing at a height of ~50 cm above the soil, which the lizards could not climb. Enclosures had open tops and were separated from one another by at least 5m. In high-risk [FA+] enclosures we allowed fire ant mounds to persist at natural densities (a mean of 7 mounds per enclosure, from 10 counts carried out during the experiment). In low-risk [FA–] enclosures, we treated fire ant mounds with Amdro (Central Garden & Pet Company, Atlanta, GA), a targeted insecticide that is taken by the ants directly into the mound as food ensuring a highly localized treatment (Darracq et al. 2017) one week prior to offspring release. Fire ant mound density remained close to zero for the duration of the experiment following initial treatment. Since fence lizards show a strong learned aversion to the consumption of fire ants as juveniles (Venable et al. 2019) we do not expect that removing fire ant mounds reduced food availability. Grass along enclosure edges was kept short (cut manually with small clippers) throughout the experiment to ensure lizards were unable to climb out. All enclosures had similar availability of shelter and shade and were constructed on similar terrain. To provide additional refugia, and to serve as standardized release sites, two wooden pallets (approx. 1x1x0.1 m) were stacked at three points within each enclosure.

Four offspring from each mother were randomly assigned to each of the four enclosures, such that each enclosure contained one offspring from each clutch, and each clutch contributed offspring to both high- and low-fire ant predation risk enclosures, in duplicate. A total of 121 offspring were released into the four enclosures: 56 from CORT-treated mothers (27 in FA– enclosures, 29 in FA+ enclosures), and 65 from control mothers (30 in FA– enclosures, 33 in FA+ enclosures). Each offspring was given an individually identifying toe clip for permanent identification and was color-coded with two small dots of non-toxic nail polish (Pure Ice, New York) on the dorsal surface to allow visual identification from a distance. A previous study indicated that color marking did not have strong effects on survivorship in *Sceloporus* lizards (Simon and Bissinger 1983)—nevertheless, we kept markings as small as possible. Twenty-eight mothers produced enough offspring to contribute 4 offspring each to the enclosure experiment: 2 produced only 3 live offspring, 1 produced only 2, and 1 produced only one. Each of these four partial “sets” of offspring were distributed as evenly as possible across enclosures types. All offspring releases took place between 07:00 AM and 09:00 AM and within 24 hours of hatching. Releases co-occurred with a behavior assay (see “Response to a handling stressor (initial)”), following placement of the offspring on top of a pallet; offspring were not released onto pallets that had been used in the previous 2 releases. Offspring were recaptured 10 days later (or as soon as they were observed thereafter).

### Testing the effects of maternally derived glucocorticoids on offspring

We tested the effects of increased maternal glucocorticoids on offspring by measuring: 1) morphology and behavior at hatching, immediately prior to release into enclosures; 2) behavior in high or low-fire ant predation risk environments at release; and 3) growth and early-life survival in high or low-fire ant environments at 10 days old. Observers were blind to maternal treatment.

#### Maternal glucocorticoid treatment and offspring phenotype

##### Morphology

At hatching, mass (to 0.1 g), and SVL (to 0.1 mm) were measured. As these were strongly correlated, we tested maternal treatment effects only on SVL. We also tested effects on offspring body condition (residuals of the log[SVL]~log[mass] correlation).

##### Response to a handling stressor (initial)

 We tested response to a handling stressor (a common experimental stressor for reptiles (Tokarz and Summers 2011) during release into enclosures. Offspring were gently removed from transport containers and restrained by hand for 60 seconds before being released, and subsequently observed for 5 minutes. We recorded four behaviors: latency to move after handling (s), whether or not the lizard took refuge (1—took refuge, 0—did not take refuge), distance moved and elevation change (both estimated to the nearest 5 cm). Latency to move was strongly skewed towards the maximum (i.e. few individuals moved at all), so instead, we modeled likelihood of moving (1—moved, 0—did not move).

### Statistical analysis

To test maternal glucocorticoid effects on offspring phenotype, the above measures of morphology and behavior were set as dependent variables in separate linear mixed models using the *lme4* package in R (Bates et al. 2015; R Core Team 2018). Each model included maternal treatment (control, CORT), and number of doses the mother received (to account for variation in the duration of maternal treatment) as categorical independent variables, as well as an interaction term (maternal treatment * dose number), to test if duration of treatment had variable effects across the control and CORT treatments. In models testing effects on behavior, we also included SVL at hatching as an independent variable. Clutch ID was included as a random term in all models to account for non-independence of data from siblings. Binomial model structures were used where data were binary (i.e. refuge sought or not).

#### Offspring behavior in high and low-fire ant predation risk environments

##### Detection probability/activity

A census of each enclosure was conducted every day between 07:00 AM and 10:00 AM. Prior to census, temperature (C˚) and cloud cover (%, estimated visually) were recorded and then one person walked slowly through each enclosure following established grid lines (each 20 m edge divided into eight 2.5 m^2^ grids), scanning trees, pallets, and the ground for offspring. Once a lizard was seen, the researcher would slowly approach until they could recognize the color code using binoculars: handling was not required in order to identify lizards. Offspring ID, time of day, type of substrate (tree [>5cm above ground], pallet, ground), and grid reference point were recorded. Offsprings not seen during the census were noted as absent. The order in which enclosures were censused was randomized every day, and only one researcher censused an enclosure to ensure equal sampling effort. The majority of censuses were conducted by the lead author; on a small number of occasions one of two trained field assistants conducted censuses. Using these data we quantified detection probability (a proxy for activity: Downes and Hoefer 2004)).

##### Range size

. Grid reference points measured during the daily census allowed us to calculate the Mean Maximum Distance Moved (Otis et al. 1978), the average distance between detections of a single individual (with “maximum distance” referring to the greatest distance between any two capture points), a proxy for range size (Karanth et al. 2006; Püttker et al. 2012; Tobler and Powell 2013), using the R package *secr* (Efford 2019). We limited the dataset to individuals that were detected at least 4 times, which led to the exclusion of 30 individuals of the original 121 released (*N* = 91).

##### Elevated basking and proximity to refugia

. We binned the substrate type on which offspring were found into two categories: elevated basking sites (open areas >5 cm from ground level suitable for basking: e.g., side or top of pallet, tree) and close to ground (</=5 cm from the ground e.g., under pallet, on ground). We mapped refugia (pallets and trees) onto our grid system, allowing us to calculate from grid reference sightings whether or not offspring were found in grids containing refugia or not. The number of days offspring were seen elevated, on the ground, and in grids in the open or containing refugia were separately calculated. In both cases, data were combined into bound columns using the *cbind* function in R (sightings elevated vs near ground, and sightings near refugia vs sightings in the open).

##### Response to a handling stressor (post-enclosure)

. Handling stressor trials were conducted a second time when offspring were captured for removal from enclosures (day 10 or 11). Lizards were caught and held in the hand for 60s prior to being re-released at the point of capture and observed for 5 minutes following the protocol described in section “Response to a handling stressor (initial)”. Offspring not located on day 10 or 11 (*N* = 15 of the ultimate *N* = 98 surviving offspring) were not subject to this trial in order to standardize for age resulting in a final sample size of 83 (control = 46, CORT-treated = 37). We collected data again on the four behaviors described in section “Response to a handling stressor (initial)” (latency to move after handling, likelihood of taking refuge, distance moved during the observation, and elevation change). Individuals who did not move during the trial (*N* = 9) were given a maximum score of 300 seconds for latency to move, instead of excluding them. Offspring were recaptured following the trial and removed from enclosures.

### Statistical analysis

We estimated the effects of maternal and enclosure treatment on offspring detection probability (and survival—see section “Early life survival”) using a Cormack-Jolly-Seber survival model in Program MARK (White and Burnham 1999). This type of survival model is especially useful in ecological studies as it provides estimates of “capture rates” (i.e. detection probability) from capture-recapture data. A global model estimated detection probability (*p,* the probability that an offspring that was present was detected during a daily census) according to maternal treatment and offspring fire ant predation risk environment. Alternative models were then derived by progressive removal of factors. All potential alternative models were tested and compared using QAICc. Global model fit to the data was assessed using Fletcher c-hat (Fletcher 2012).

To test treatment effects on offspring behavior in high and low-fire ant environments, the above measures of range size, substrate use, and behavior were set as dependent variables in separate linear mixed models. Detection probability/activity were analysed separately as described. Each model contained the following independent variables: maternal treatment and offspring fire ant predation risk environment (FA+, FA–) as an interaction term, maternal treatment and dose number as a separate interaction term, and SVL (cm) at hatching. Clutch ID and enclosure number were included as random terms in all models to account for non-independence of data from siblings, and within enclosures. Binomial model structures were used when data were bound columns (i.e. days elevated, days not elevated) as this functions as proportion data which is bound at 0 and 1.

#### Maternal treatment and offspring fitness measures in high and low fire ant predation risk environments

##### Growth and body condition

. We re-measured all surviving offspring upon removal from enclosures. We calculated a growth metric (daily increase in body size, calculated as the difference between ultimate SVL and SVL at hatching, divided by the age in days at ultimate measurement). We excluded 7 surviving offspring that were found when older than 15 days (sample *N* = 91). SVL, body condition, and growth rate were set as dependent variables in separate LMMs, again containing the following variables: maternal treatment and offspring fire ant environment (FA+, FA–) as an interaction term, maternal treatment and dose number as a separate interaction term, and SVL (cm) at hatching with maternal treatment and offspring fire ant environment as an interaction term, and clutch and enclosure ID as random terms.

##### Early-life survival

. We estimated the effects of maternal treatment and enclosure fire ant predation risk level (FA+/FA–) on early-life survival using a Cormack-Jolly-Seber survival analysis, the same model as described in section “Detection probability/activity”. A global model estimated survival *phi* according to maternal treatment and offspring fire ant environment, mass, SVL, and mean density (mean number of individuals present in the enclosure over the 10 day period that an offspring was in the enclosure). Alternative models were then derived by progressive removal of factors and compared using QAICc (full candidate model set reported in Supplementary Table S3).

## RESULTS

Full model results are reported in Supplementary Material S1. If not reported below, terms were not significant at *P* > 0.05.

### Maternal glucocorticoid treatment and offspring phenotype

#### Morphology

There was a non-significant trend for offspring SVL at hatching to decrease as maternal treatment duration increased in the CORT treatment group (rho –0.26) while SVL increased as maternal treatment duration increased in the control group (rho 0.3; maternal treatment*dose number interaction *X*^2^_1,118_ = 3.30, *P* = 0.07). Maternal treatment alone did not influence offspring SVL (*X*^2^_1,119_ = 0.01, *P* = 0.91). Maternal CORT treatment had a positive but non-significant effect on offspring body condition at hatching: offspring from CORT-treated mothers tended to be relatively heavier for their size compared to those from control mothers (*X*^2^_1,119_ = 3.29, *P* = 0.07).

#### Offspring response to a handling stressor

Maternal CORT treatment did not affect how offspring responded to a handling stressor; there were no effects on latency to move (*X*^2^_1,120_ = 0.18, *P* = 0.69); likelihood of taking refuge (*X*^2^_1,118_ = 0.15, *P* = 0.69); distance moved (*X*^2^_1,118_ = 2.40, *P* = 0.12); or change in elevation (*X*^2^_1,120_ = 1.16, *P* = 0.28).

### Offspring behavior in high- and low-fire ant predation risk environments

#### Detection probability/activity

**Figure 1:**
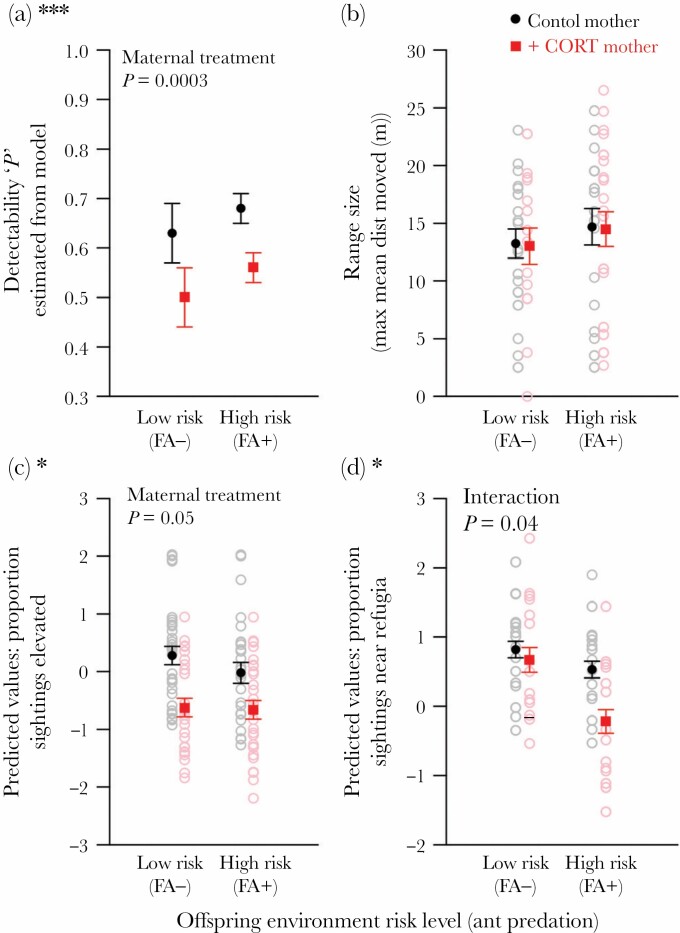
Offspring a) activity [‘p’, detection probability estimate from MARK recapture model]; b) range size [Mean Max Distance Moved]; c) proportion of sightings on elevated substrate (i.e. basking; predicted values from model); and d) proportion of sightings near shelter (in same grid; predicted values from model) throughout the trial. Offspring were reared either in low-risk (no fire ants present, FA–) or high-risk (high density of fire ants, FA+) enclosures from one to 10/11 days of age. Offspring from control mothers (*N* = 65) shown in black; offspring from CORT-treated mothers (*N* = 56) shown in red.

Maternal CORT treatment had a negative effect on offspring detection probability *p* (Figure 1a). In two models, the best and third best models according to AIC (Table 1a,c), *p* was strongly predicted by maternal treatment (top model: *X*^2^_1,120_ = 12.97, *P* = 0.0003), with offspring from control mothers significantly more likely to be detected during census than offspring from CORT-treated mothers across both fire ant environments. In the second best model according to AIC (Table 1b), detection probability was predicted by an interaction of maternal treatment and offspring fire ant environment (*X*^2^_1,118_ = 15.47, *P* = 0.0015) describing increased ant predation risk in early life having a positive effect on detection probability, with offspring from control mothers in FA+ enclosures being the most detectable (estimate 0.68).

**Table 1:** Estimates for survival (*phi*) and detection probability (*p*) parameters for all models within 2AIC of the best model (according to Akaike Information Criterion)

	Model	ΔAIC	Parameter	Estimate	SE	X^2^	P
a)	phi(.) p(maternal treatment)	0	*phi (null)*	*0.97*	*0.005*		
			Maternal treatment			12.97	0.0003
			p_CORT_	0.53	0.02		
			p_control_	0.65	0.02		
b)	phi(.) p(maternal/offspring risk environment)	1.55	*phi (null)*	0.97	0.005		
			Maternal/offspring risk environment			15.47	0.0015
			p_CORT*hi-pred_	0.56	0.03		
			p_CORT*low-pred_	0.50	0.06		
			p_control*hi-pred_	0.68	0.03		
			p_control*low-pred_	0.63	0.03		
c)	phi(maternal treatment) p(maternal treatment)	1.63	Maternal treatment			0.39	0.53
			phi_CORT_	0.98	0.007		
			phi_control_	0.97	0.006		
			Maternal treatment			13.33	0.0003
			p_CORT_	0.53	0.02		
			p_control_	0.65	0.02		

*P* values calculated from Likelihood Ratio Tests testing the model of interest against a null model for that parameter.

#### Range size

We found no effects on offspring range size (the mean maximum distance between each individual sighting) of maternal CORT treatment (*X*^2^_1,89_ = 0.45, *P* = 0.50), offspring fire ant environment (*X*^2^_1,89_ = 0.60, *P* = 0.44), and no interaction effects (*X*^2^_1,89_ = 2.12, *P* = 0.35) (Figure 1b).

#### Elevated substrate use, and proximity to refugia

Offspring from CORT-treated mothers were more likely to be found on substrates lower to the ground than offspring from control mothers (*X*^2^_1,108_ = 3.76, *P* = 0.05; Table 2a) regardless of ant predation risk level (i.e. no effect of offspring environment [*X*^2^_1,108_ = 0.40, *P* = 0.52] and no interaction effect [*X*^2^_1,107_ = 0.85, *P* = 0.66; Figure 1c]).

**Table 2:** Results from generalised linear mixed models (GLMMs) showing significant effects of maternal treatment alone, or in interaction with offspring risk environment (level of fire ant predation). Where the interaction was significant, results of Tukey post-hoc comparisons are also shown

Model		Predictor	Estimate	SE	Z	P
a)	Likelihood of being found on elevated substrate	*Intercept*	*0.19*	*0.31*	*0.61*	
		Maternal treatment				0.05 *
		control				
		CORT	–0.83	0.42	–1.99	
b)	Likelihood of being found near refugia	*Intercept*	*0.82*	*0.29*	*2.82*	
		Maternal treatment – CORT	–0.07	0.44	–0.16	
		Offspring risk environment – FA+	–0.18	0.26	–0.72	
		Maternal treatment * Offspring risk environment	–0.80	0.40	–2.02	0.04 *
		*control,FA– - CORT,FA-*	*0.07*	*0.44*		*0.99*
		*control,FA– - control,FA+*	*0.18*	*0.26*		*0.89*
		*control,FA– - CORT,FA+*	*1.05*	*0.42*		0.06*
		*CORT,FA– - control,FA+*	*0.11*	*0.44*		*0.99*
		*CORT,FA– -CORT,FA+*	*0.98*	*0.30*		0.0065**
		*control,FA+ - CORT,FA+*	*0.87*	*0.43*		*0.17*
c)	Change in elevation following handling stressor test	*Intercept*	*3.55*	*1.73*	*2.05*	
		Maternal treatment – CORT	–5.57	2.48	–2.25	
		Offspring risk environment – FA+	–5.10	2.33	–2.19	
		Maternal treatment * Offspring risk environment	6.56	3.29	1.99	0.03*
		*control,FA– -CORT,FA-*	*5.57*	*2.50*		*0.08*
		*control,FA– -control,FA+*	*5.10*	*2.37*		*0.06*
		*control,FA– -CORT,FA+*	*4.11*	*2.56*		*0.86*
		*CORT,FA– -control,FA+*	*–0.47*	*2.60*		*0.60*
		*CORT,FA– -CORT,FA+*	*–1.46*	*2.64*		*0.68*
		*control,FA+ -CORT,FA+*	*–0.99*	*2.36*		0.03*

*P* values calculated from Likelihood Ratio Tests testing the model of interest against a null model for that parameter.

Offspring fire ant predation risk environment altered the influence of maternal treatment on offspring refugia proximity (*X*^2^_1,108_ = 4.10, *P* = 0.04; Table 2b; Figure 1d): offspring from CORT-treated mothers in FA+ enclosures were less likely to be found near a refuge site compared to offspring from either treatment living in low-risk FA– enclosures (Tukey post-hoc comparisons: offspring of CORT-treated mothers *P* = 0.006; offspring of control mothers *P* = 0.06), whereas this effect was not observed for offspring from control mothers (Tukey post-hoc comparisons: offspring of CORT-treated mothers *P* = 0.99; offspring of control mothers *P* = 0.89). All other post-hoc contrasts were non-significant.

#### Juvenile response to a handling stressor, day 10

**Figure 2:**
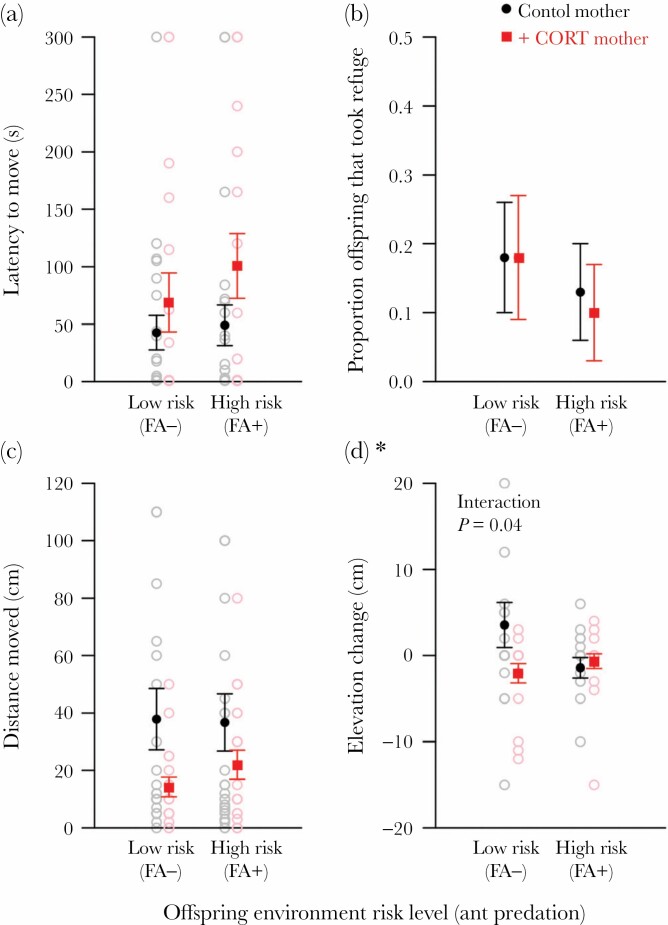
Offspring behavior following a handling stressor at day 10 of the trial: a) latency to move; b) proportion offspring that sought refuge; c) distance moved; d) elevation change. Offspring from control mothers (*N* = 46) shown in black; offspring from CORT-treated mothers (*N* = 37) shown in red. Offspring were reared either in low-risk (no fire ants present, FA–) or high-risk (high density of fire ants, FA+) enclosures from one to 10/11 days of age.

We found no effect on the latency of offspring to move after a handling stressor of maternal CORT treatment (*X*^2^_1,80_ = 2.13, *P* = 0.14), offspring environment (*X*^2^_1,80_ = 1.30, *P* = 0.25), or interaction between these two factors (*X*^2^_1,79_ = 0.40, *P* = 0.53) (Figure 2a). Larger individuals (greater SVL) had a lower latency to move (*X*^2^_1,82_ = 4.5, *P* = 0.03). So few individuals took refuge after a handling stressor at day 10 (N = 12) that modelling without singular fit was not possible: from CORT-treated mothers, 3 offspring in FA– enclosures and 2 in FA+ enclosures took refuge; from control mothers, 4 offspring in FA– enclosures and 3 in FA+ enclosures took refuge (Figure 2b). We found no effect on the distance offspring moved after a handling stressor of maternal CORT treatment (*X*^2^_1,79_ = 1.01, *P* = 0.31), offspring environment (*X*^2^_1,79_ = 0.12, *P* = 0.90), or interaction effects (*X*^2^_1,78_ = 0.04, *P* = 0.83) (Figure 2c). Distance moved increased with offspring SVL (*X*^2^_1,79_ = 5.56, *P* = 0.02). Offspring fire ant risk environment altered the influence of maternal treatment on elevation change (*X*^2^_1,80_ = 4.15, *P* = 0.04; Table 2c; Figure 2d); offspring from control mothers moved higher after handling compared to offspring from CORT-treated mothers in low-risk (FA–) environments (Tukey post-hoc comparison, *P* = 0.03). No other contrasts were statistically significant.

### Maternal treatment and offspring fitness measures in high and low-fire ant predation risk environments

#### Growth and body condition, day 10

There were no effects of maternal treatment on offspring body condition (*X*^2^_1,80_ = 0.16, *P* = 0.69) or SVL at day 10 (*X*^2^_1,80_ = 0.44, *P* = 0.51). Neither were there effects of offspring environment on body condition (*X*^2^_1,80_ = 2.49, *P* = 0.11) or SVL (*X*^2^_1,80_ = 0.00, *P* = 0.99); or interactive effects of maternal treatment and offspring environment on body condition (*X*^2^_1,80_ = 1.32, *P* = 0.25) or SVL (*X*^2^_1,80_ = 0.03, *P* = 0.86). Offspring growth was not affected by maternal treatment (*X*^2^_1,80_ = 1.25, *P* = 0.26), offspring ant predation risk environment (*X*^2^_1,80_ = 0.59, *P* = 0.49); or interactive effects (*X*^2^ = 0.48, *P* = 0.44). Maternal treatment duration correlated negatively with growth (dose number *X*^2^_1, 80_ = 5.18, P = 0.02); this effect did not depend on treatment type (dose number*treatment *X*^2^_1, 79_ = 0.33, *P* = 0.54). Originally larger offspring grew more slowly (SVL at hatching *X*^2^_1, 80_ = 7.35, *P* = 0.007).

#### Early-life survival

A total of 98 offspring were recaptured from enclosures at day 10 or as soon as sighted thereafter. Offspring from control mothers had 93.8% survival in FA– enclosures and 75.8% survival in FA+ enclosures (55 survivors of original *N* = 65). Offspring from CORT-treated mothers had 77.8% in FA– enclosures and 75.9% survival in FA+ enclosures (43 survivors of original *N* = 56). Neither maternal CORT treatment nor offspring fire ant predation risk environment significantly explained offspring survival to day 10; a fixed effect was better than a null slope in only one of the three best MARK models using census data (ΔQAICc <2 of top model), and in this case it was non-significant (maternal treatment, *X*^2^ = 0.39, *P* = 0.53; Table 1c).

## DISCUSSION

Increased maternal glucocorticoids during gestation had limited effects on offspring morphology and behavior at birth in eastern fence lizards. There was, however, indication that prolonged glucocorticoid elevation had the potential for effects on morphology: in the CORT treatment group, offspring body size decreased with maternal treatment duration, while in the control group, offspring size increased with treatment duration. The latter is likely due to increased capacity to invest in offspring under captive conditions where food and water availability is constant (MacLeod et al. 2018b). However, after 10 days, differences became apparent according to maternal treatment in offspring predator avoidance behaviors, including some that were dependent on the level of risk of fire ant predation in the environment in which the offspring were raised/living. Specifically, offspring from CORT-treated mothers reduced basking and spent less time on elevated substrates overall; spent less time near refugia in high-ant risk enclosures compared to all offspring in low-risk enclosures; and had reduced detectability/activity levels, particularly in low-ant risk environments. These results suggest that the phenotypic outcomes of glucocorticoid-driven maternal effects are at least somewhat context-dependent, and in this case, are influenced by the offspring’s early life risk environment.

Our results add to a growing body of literature suggesting that maternal glucocorticoids are a likely mechanistic link in the induction of antipredator phenotypes in offspring (De Fraipont et al. 2003; Uller and Olsson 2006; Robert et al. 2009), and may therefore have the potential to provide adaptive benefit to the next generation in line with anticipatory maternal effects theory (Marshall and Uller 2007). Offspring from CORT-treated mothers could be better adapted to a high-risk environment: in wild lizard systems, reduced activity and reduced time on elevated substrates lessen rates of detection by and encounters with predators, particularly birds (Downes and Shine 1999, 2000). However, while avoiding elevated substrates and reducing activity may adaptively reduce visibility to aerial predators, there is a higher risk of fire ant predation on the ground (Langkilde 2009a). This therefore could alternatively be an example of an “evolutionary trap”—cue-response systems adaptive under historic conditions result in maladaptive behaviors under novel selective pressures in changed environments (Donelan et al. 2020). A previous study of behavioral adaptation to fire ant predation in eastern fence lizard populations showed a link between anti-ant behavior and increased injury rates, providing further evidence that aerial and ant predation might indeed exert opposing selective pressures in this system, with adaptations to one (driven by novel threat posed by fire ants) resulting in increased threat from the other (Thawley and Langkilde 2017). Unfortunately linking offspring behavior to predation rates directly was beyond the scope of this study but would be a useful next step in linking phenotypic changes invoked by the prenatal environment with potentially adaptive—or perhaps maladaptive—outcomes in this species.

In cases where multiple predator types select for opposing traits in offspring, glucocorticoids as a general “ecological integrator” linking maternal and offspring predator environments may have reduced efficacy as they are likely to only convey information about level, not type, of risk. We did not see increased mortality of individuals based on offspring risk environment (fire ant predation). It therefore seems likely that, at least at this life history stage, selection for anti-ant behavior is not strong enough to override the “general rules” of antipredator behavior (Orr 2000). Generalized antipredator behaviors (such as reduced activity levels) most often lower predation risk in the presence of multiple predators (Sih et al. 1998), and are likely to be easier to evolve and maintain than species-specific responses (Blumstein 2006). A number of lizard species display generalised antipredator responses to snake cues regardless of threat level (Stapley 2003; Amo et al. 2004; Webb et al. 2009). Here, responding to a harmless snake cue is unlikely to be as costly as the risk of misidentifying a predatory snake, thus the behavior is maintained. Similarly, it is potentially adaptive to retain generalised antipredator responses even at the risk of increasing encounter rates with ant predators given the difference in lethality in encounters with these two predators (Sheriff et al. 2020). Selection imposed by avian predation is likely stronger due to relative higher lethality of bird-lizard encounters, resulting in the maintenance of an anti-bird behavioral phenotype, while lizards can still avoid being killed by fire ants by responding even after they have been attacked (Langkilde 2009b; Trompeter and Langkilde 2011b). As fire ants are more likely to find and attack lizards in shelter than on elevated surfaces (Langkilde 2009a), we would expect that shelter would be avoided in these environments. However, a species-specific behavior like shelter-avoidance may be unlikely to evolve or be maintained when shelter-use likely confers general adaptive benefits (Martín and López 1999; Amo et al. 2007). Early life experience may serve to effectively refine the generalist antipredator phenotype induced by maternal glucocorticoids; for example, we saw decreased shelter use by offspring from CORT-treated mothers in high ant-risk enclosures.

Despite this tentative evidence that maternal CORT treatment induced antipredator behavior in offspring, we found no effects on early-life survival, and this was not dependent on the offspring’s early life fire ant predation risk environment. We therefore cannot designate the effects seen on behavior as anticipatory maternal effects, or adaptive phenotypic plasticity, based on these results (Marshall and Uller 2007). Although an important period of offspring vulnerability to predation (Parker 1994) making this a likely cause for the mortality that we did see, it is likely that we did not see sufficient mortality in the first 10 days of life to quantify meaningful variation in survival between treatment groups. This period (10 days) was based on reports of low juvenile survival in the first weeks post-hatching, and survival in a pilot study (39% survival to day 10, *N* = 54). Here, the difference between survival of offspring from control and CORT-treated mothers was not significant (a ~15% difference in survival represented a difference of only 5 individuals (i.e. 2 vs 7 deaths). Similarly, there were no apparent consequences of behavioral variation among treatment groups on offspring growth, though behavioral changes associated with high risk (such as decreased activity) often trades off with activities important for energy intake and growth, such as foraging and basking (Huey 1982; Downes 2001).

To our knowledge this is among the first studies to examine, in a semi-natural setting using wild animals, potential interactive effects between prenatal glucocorticoid exposure and variation in predation risk from a key predator in the offspring’s early environment. Our results highlight the importance of testing maternal effects across time periods, and critically, across contexts and natural environments (Sheriff et al. 2017) as maternal CORT can affect how offspring respond to stressors in their environment. We suggest that maternal CORT effects may prepare offspring for historical threats and thus may maladapt them to new environmental threats (Sheriff et al. 2018; Donelan et al. 2020). Repeating this experiment using predator-specific cues would help to test the specificity of this response. Further, testing how these maternal effects may change over evolutionary time to better prepare offspring for the specific characteristics of “matched” high-stressor environments (e.g. high temperatures, food limitation, introduced predators) would shed light on the adaptive significance of this phenomenon.

## Supplementary Material

arab099_suppl_Supplementary_Material

## Data Availability

Analyses reported in this article can be reproduced using the data provided by MacLeod (2021).
