# Supplementary material for: The influence of maternal glucocorticoids on offspring phenotype in high- and low-risk environments
Source: Behav Ecol. 2021 Sep 10;32(6):1330–8. doi: 10.1093/beheco/arab099 (PMC8691550; doi:10.1093/beheco/arab099)
Supplement: arab099_suppl_Supplementary_Material [file arab099_suppl_supplementary_material.docx]

Supplementary material

**S1. Full results of all models. Significance of fixed effects determined in separate model without interaction as specified.**

- 1. Morphology (N = 121 [65 control, 56 CORT], 32 clutches)
     1. *SVL* ~ treatment*dose number + [1|clutch ID]/treatment + dose number + [1|clutch ID]

| **predictor** | **est.** | **s.e.** | **T** | **P** |
| --- | --- | --- | --- | --- |
| *Intercept* | *2.30* | *0.06* | *38.78* |  |
| Treatment * dose number | -0.02 | 0.01 | -1.74 | 0.07 |
|  |  |  |  |  |
| *Intercept* | *2.38* | *0.04* | *56.02* |  |
| Treatment (CORT) | -0.005 | 0.04 | -0.11 | 0.91 |
| Dose number | -0.0004 | 0.004 | -0.008 | 0.99 |

Interaction - strong trend towards a positive effect of dose number on SVL in control group (*Spearman’s rho* 0.3, P = 0.01) and a negative effect of dose number on SVL in CORT group (*Spearman’s rho* -0.26, P = 0.05).

- - 1. *Body condition* ~ treatment*dose number + [1|clutch ID]/treatment + dose number + [1|clutch ID]

| **predictor** | **est.** | **s.e.** | **T** | **P** |
| --- | --- | --- | --- | --- |
| *Intercept* | *0.02* | *0.05* | *0.38* |  |
| Treatment * dose number | 0.01 | 0.008 | 0.74 | 0.43 |
|  |  |  |  |  |
| *Intercept* | *-0.008* | *0.03* | *-0.23* |  |
| Treatment (CORT) | 0.06 | 0.03 | 1.77 | 0.07 |
| Dose number | -0.002 | 0.004 | -0.61 | 0.53 |

- 1. Hatchling response to a handling stressor (N = 121 [65 control, 56 CORT], 32 clutches)
     1. *Latency to move* ~ treatment*dose number + svl + [1|clutch ID]/treatment + dose number + svl + [1|clutch ID]

| **predictor** | **est.** | **s.e.** | **T** | **P** |
| --- | --- | --- | --- | --- |
| *Intercept* | *0.02* | *3.90* | *0.004* |  |
| Treatment * dose number | -0.02 | 0.11 | -0.21 | 0.83 |
| SVL | 0.39 | 1.67 | 0.24 | 0.81 |
|  |  |  |  |  |
| *Intercept* | *-0.11* | *3.85* | *-0.03* |  |
| Treatment (CORT) | -0.56 | 0.44 | -1.28 | 0.69 |
| Dose number | 0.01 | 0.05 | 0.20 | 0.25 |
| SVL | 0.49 | 1.61 | 0.31 | 0.76 |

- - 1. *Likelihood of taking refuge* ~ treatment*dose number + svl + [1|clutch ID]/treatment + dose number + svl + [1|clutch ID]

| **predictor** | **est.** | **s.e.** | **Z** | **P** |
| --- | --- | --- | --- | --- |
| *Intercept* | *-3.87* | *5.46* | *-0.71* |  |
| Treatment * dose number | -0.32 | 0.17 | -1.95 | 0.06 |
| SVL | 0.001 | 2.29 | 0.001 | 0.99 |
|  |  |  |  |  |
| *Intercept* | *-5.33* | *5.62* | *-0.95* |  |
| Treatment (CORT) | -0.27 | 0.67 | -0.40 | 0.69 |
| Dose number | 0.09 | 0.08 | 1.18 | 0.25 |
| SVL | 1.22 | 2.31 | 0.53 | 0.60 |

(N.B. AIC improved by removing interaction)

- - 1. *Distance moved*~ treatment*dose number + svl + [1|clutch ID]/treatment + dose number + svl + [1|clutch ID]

| **predictor** | **est.** | **s.e.** | **T** | **P** |
| --- | --- | --- | --- | --- |
| *Intercept* | *1.43* | *3.24* | *0.44* |  |
| Treatment * dose number | -0.05 | 0.09 | -0.57 | 0.56 |
| SVL | 0.30 | 1.39 | 0.21 | 0.83 |
|  |  |  |  |  |
| *Intercept* | *1.12* | *3.18* | *0.35* |  |
| Treatment (CORT) | -0.56 | 0.37 | -1.53 | 0.12 |
| Dose number | 0.03 | 0.04 | 0.80 | 0.41 |
| SVL | 0.52 | 1.33 | 0.39 | 0.69 |

- - 1. *Change in elevation* ~ treatment*dose number + svl + [1|clutch ID]/treatment + dose number + svl + [1|clutch ID]

| **predictor** | **est.** | **s.e.** | **T** | **P** |
| --- | --- | --- | --- | --- |
| *Intercept* | *-8.11* | *17.11* | *-0.47* |  |
| Treatment * dose number | 0.26 | 0.51 | 0.51 | 0.58 |
| SVL | 2.37 | 7.32 | 0.32 | 0.74 |
|  |  |  |  |  |
| *Intercept* | *-6.80* | *16.78* | *-0.41* |  |
| Treatment (CORT) | 2.08 | 2.02 | 1.03 | 0.28 |
| Dose number | -0.23 | 0.23 | -0.97 | 0.29 |
| SVL | 1.37 | 6.99 | 0.20 | 0.85 |

## c) Offspring behaviour in high- and low-risk environments

i) Detection probability/activity (N = 121 [65 control, 56 CORT], 32 clutches)

(see 3b for full set of MARK models; best model(s) reported in manuscript main text)

ii) Range size (N = 101 [49 control, 52 CORT])

- Mean Max Dist ~ Treatment:Enclosure_type+Treatment:DoseNum + Treatment+Enclosure_type+DoseNum+SVL + (1|clutchID)+ (1|enclosure)
- Mean Max Dist ~ Treatment+Enclosure_type+DoseNum+SVL + (1|clutchID)+ (1|enclosure)

| **predictor** | **est.** | **s.e.** | **T** | **P** |
| --- | --- | --- | --- | --- |
| *Intercept* | *0.81* | *1.88* | *0.43* |  |
| Treatment * dose number | -0.03 | 0.06 | -0.56 | 0.56 |
| Treatment * enclosure type | 0.42 | 0.32 | 1.29 | 0.21 |
| SVL | 0.56 | 0.80 | 0.70 | 0.45 |
|  |  |  |  |  |
| *Intercept* | *1.00* | *1.82* | *0.55* |  |
| Treatment (CORT) | -0.15 | 0.23 | -0.65 | 0.50 |
| Enclosure type (FA+) | 0.12 | 0.18 | 0.64 | 0.44 |
| Dose number | 0.02 | 0.03 | 0.82 | 0.40 |
| SVL | 0.50 | 0.75 | 0.67 | 0.46 |

iii) Elevation/basking and proximity to refugia (N = 101 [58 control, 52 CORT])

*Likelihood of being found on elevated substrate*

- ~ Treatment:Enclosure_type+Treatment:DoseNum + Treatment+Enclosure_type+DoseNum+SVL + (1|clutchID)+ (1|enclosure)
- ~ Treatment+Enclosure_type+DoseNum+SVL + (1|clutchID)+ (1|enclosure)
- ~ Treatment + (1|clutchID)+ (1|enclosure)

| **predictor** | **est.** | **s.e.** | **T** | **P** |
| --- | --- | --- | --- | --- |
| *Intercept* | *2.30* | *2.94* | *0.78* |  |
| Treatment * dose number | 0.17 | 0.11 | 1.56 | 0.13 |
| Treatment * enclosure type | 0.29 | 0.40 | 0.73 | 0.47 |
| SVL | -0.44 | 1.24 | -0.36 | 0.72 |
|  |  |  |  |  |
| *Intercept* | *2.94* | *2.94* | *1.00* |  |
| Treatment (CORT) | -0.75 | 0.44 | -1.69 | 0.09 |
| Enclosure type (FA+) | -0.19 | 0.30 | -0.65 | 0.52 |
| Dose number | -0.02 | 0.05 | -0.47 | 0.64 |
| SVL | -1.05 | 1.21 | -0.87 | 0.39 |
|  |  |  |  |  |
| *Intercept* | *0.19* | *0.31* | *0.61* |  |
| Treatment (CORT) | -0.83 | 0.42 | -1.99 | 0.05 * |

*Likelihood of being found near refugia*

- ~ Treatment:Enclosure_type+Treatment:DoseNum + Treatment+Enclosure_type+DoseNum+SVL + (1|clutchID)+ (1|enclosure)
- ~ Treatment * Enclosure_type

| **predictor** | **est.** | **s.e.** | **Z** | **P** |
| --- | --- | --- | --- | --- |
| *Intercept* | *1.09* | *2.85* | *0.38* |  |
| Treatment * dose number | 0.12 | 0.10 | 1.17 | 0.24 |
| Treatment * enclosure type | -0.81 | 0.40 | -1.99 | 0.04 * |
| SVL | 0.23 | 1.21 | 0.20 | 0.85 |
|  |  |  |  |  |
| *Intercept* | *0.82* | *0.29* | *2.82* |  |
| Treatment * enclosure type | -0.80 | 0.40 | -2.02 | 0.04* |

Tukey post-hoc test of model results testing likelihood of being found near refugia: maternal treatment * enclosure risk level significant (P = 0.04*).

| **contrast** | **estimate** | **s.e.** | ***P*** |
| --- | --- | --- | --- |
| control,FA- - CORT,FA- | 0.07 | 0.44 | 0.99 |
| control,FA- - control,FA+ | 0.18 | 0.26 | 0.89 |
| control,FA- - CORT,FA+ | 1.05 | 0.42 | 0.06* |
| CORT,FA- - control,FA+ | 0.11 | 0.44 | 0.99 |
| CORT,FA- - CORT,FA+ | 0.98 | 0.30 | 0.0065** |
| control,FA+ - CORT,FA+ | 0.87 | 0.43 | 0.17 |

d) Juvenile response to a handling stressor (N = 83 [46 control, 37 CORT])

*i) Latency to move*

- ~ Treatment:Enclosure_type+Treatment:DoseNum + Treatment+Enclosure_type+DoseNum+SVL + (1|clutchID)+ (1|enclosure)
- ~ Treatment+Enclosure_type+DoseNum+SVL + (1|clutchID)+ (1|enclosure)

| **predictor** | **est.** | **s.e.** | **T** | **P** |
| --- | --- | --- | --- | --- |
| *Intercept* | *463.44* | *203.06* | *2.28* |  |
| Treatment * dose number | 6.96 | 7.12 | 0.98 | 0.30 |
| Treatment * enclosure type | 23.31 | 37.71 | 0.62 | 0.53 |
| SVL | -148.5 | 74.85 | -1.98 | 0.03 * |
|  |  |  |  |  |
| *Intercept* | *443.48* | *201.83* | *2.19* |  |
| Treatment (CORT) | 40.33 | 28.39 | 1.42 | 0.14 |
| Enclosure type (FA+) | 22.92 | 23.43 | 0.98 | 0.25 |
| Dose number | -0.77 | 3.43 | -0.23 | 0.84 |
| SVL | -153.51 | 74.44 | -2.06 | 0.03 * |

- - 1. *Likelihood of taking refuge* - Too few took refuge to analyse meaningfully.
    2. *Distance moved*
- ~ Treatment:Enclosure_type+Treatment:DoseNum + Treatment+Enclosure_type+DoseNum+SVL + (1|clutchID)+ (1|enclosure)
- ~ Treatment+Enclosure_type+DoseNum+SVL + (1|clutchID)+ (1|enclosure)

| **predictor** | **est.** | **s.e.** | **T** | **P** |
| --- | --- | --- | --- | --- |
| *Intercept* | *-2.83* | *2.74* | *-1.03* |  |
| Treatment * dose number | -0.03 | 0.09 | -0.37 | 0.68 |
| Treatment * enclosure type | 0.10 | 0.52 | 0.20 | 0.83 |
| SVL | 2.23 | 1.01 | 2.20 | 0.03 * |
|  |  |  |  |  |
| *Intercept* | *-2.80* | *2.70* | *-1.04* |  |
| Treatment (CORT) | -0.36 | 0.36 | -0.97 | 0.31 |
| Enclosure type (FA+) | 0.02 | 0.39 | 0.06 | 0.90 |
| Dose number | -0.05 | 0.04 | -1.08 | 0.23 |
| SVL | 2.26 | 1.00 | 2.27 | 0.03 * |

- - 1. *Change in elevation*
- ~ Treatment:Enclosure_type+Treatment:DoseNum + Treatment+Enclosure_type+DoseNum+SVL + (1|clutchID)+ (1|enclosure)
- ~ Treatment * Enclosure_type

| **predictor** | **est.** | **s.e.** | **Z** | **P** |
| --- | --- | --- | --- | --- |
| *Intercept* | *11.78* | *15.87* | *0.74* |  |
| Treatment * dose number | -0.65 | 0.49 | -1.34 | 0.15 |
| Treatment * enclosure type | 7.47 | 3.36 | 2.22 | 0.03 * |
| SVL | -4.38 | 5.87 | -0.75 | 0.42 |
|  |  |  |  |  |
| *Intercept* | *3.55* | *1.73* | *2.05* |  |
| Treatment * enclosure type | 6.56 | 3.29 | 1.99 | 0.04* |

**Tukey post-hoc test of model results testing change in elevation after handling stressor: maternal treatment * enclosure risk level significant (P = 0.04*).**

| **contrast** | **estimate** | **s.e.** | ***P*** |
| --- | --- | --- | --- |
| control,FA- - CORT,FA- | 5.57 | 2.50 | 0.03* |
| control,FA- - control,FA+ | 5.10 | 2.37 | 0.08 |
| control,FA- - CORT,FA+ | 4.11 | 2.56 | 0.06 |
| CORT,FA- - control,FA+ | -0.47 | 2.60 | 0.86 |
| CORT,FA- - CORT,FA+ | -1.46 | 2.64 | 0.60 |
| control,FA+ - CORT,FA+ | -0.99 | 2.36 | 0.68 |

## e) Maternal treatment and offspring fitness measures in high and low risk environments

i) Growth and body condition (N = 98 [55 control, 43 CORT])

*SVL*

- ~ Treatment:Enclosure_type+Treatment:DoseNum + Treatment+Enclosure_type+DoseNum + (1|clutchID)+ (1|enclosure)
- ~ Treatment+Enclosure_type+DoseNum + (1|clutchID)+ (1|enclosure)

| **predictor** | **est.** | **s.e.** | **T** | **P** |
| --- | --- | --- | --- | --- |
| *Intercept* | *2.68* | *0.07* | *35.75* |  |
| Treatment * dose number | -0.006 | 0.01 | -0.47 | 0.61 |
| Treatment * enclosure type | 0.007 | 0.05 | 0.13 | 0.86 |
|  |  |  |  |  |
| *Intercept* | *2.70* | *0.06* | *48.94* |  |
| Treatment (CORT) | 0.03 | 0.05 | 0.63 | 0.51 |
| Enclosure type (FA+) | -0.002 | 0.04 | -0.04 | 0.99 |
| Dose number | -0.008 | 0.006 | -1.45 | 0.13 |

*Body condition*

- ~ Treatment:Enclosure_type+Treatment:DoseNum + Treatment+Enclosure_type+DoseNum + (1|clutchID)+ (1|enclosure)
- ~ Treatment+Enclosure_type+DoseNum + (1|clutchID)+ (1|enclosure)

| **predictor** | **est.** | **s.e.** | **T** | **P** |
| --- | --- | --- | --- | --- |
| *Intercept* | *-0.02* | *0.02* | *-0.44* |  |
| Treatment * dose number | -0.003 | 0.004 | -0.86 | 0.35 |
| Treatment * enclosure type | 0.03 | 0.03 | 1.01 | 0.32 |
|  |  |  |  |  |
| *Intercept* | *-0.001* | *0.02* | *-0.03* |  |
| Treatment (CORT) | -0.004 | 0.02 | -0.25 | 0.81 |
| Enclosure type (FA+) | 0.02 | 0.01 | 1.25 | 0.21 |
| Dose number | -0.001 | 0.002 | -0.38 | 0.69 |

*Growth rate (SVL)*

- ~ Treatment:Enclosure_type+Treatment:DoseNum + Treatment+Enclosure_type+DoseNum+SVL + (1|clutchID)+ (1|enclosure)
- ~ Treatment+Enclosure_type+DoseNum+SVL + (1|clutchID)+ (1|enclosure)

| **predictor** | **est.** | **s.e.** | **T** | **P** |
| --- | --- | --- | --- | --- |
| *Intercept* | *0.20* | *0.03* | *4.05* |  |
| Treatment * dose number | 0.00 | 0.001 | 0.64 | 0.43 |
| Treatment * enclosure type | 0.002 | 0.005 | 0.46 | 0.61 |
| SVL | -0.03 | 0.01 | -2.78 | 0.006 ** |
|  |  |  |  |  |
| *Intercept* | *0.11* | *0.03* | *4.32* |  |
| Treatment (CORT) | 0.004 | 0.003 | 1.39 | 0.14 |
| Enclosure type (FA+) | 0.003 | 0.003 | 1.02 | 0.21 |
| Dose number | -0.001 | 0.0004 | -2.41 | 0.02 * |
| SVL | -0.03 | 0.01 | -3.23 | 0.002 ** |

f) Survival (N = 121 [65 control, 56 CORT], 32 clutches)

Results of top models are reported in manuscript main text.

**S2. Mean ± 1 standard error showing maternal treatment effects on offspring phenotype. All measures were taken within 24 hours of birth (see Methods).**

|  | **Offspring from CORT females** | **Offspring from control females** |
| --- | --- | --- |
| *Morphology* | *(N = 56)* | *(N = 65)* |
| SVL (cm) | 2.37 ± 0.02 | 2.38 ± 0.02 |
| Body condition | 3.10 ± 0.03 | 3.07 ± 0.02 |
| *Behavior* | *(N = 56)* | *(N = 65)* |
| Likelihood of moving (0/1) | 0.64 ± 0.06 | 0.75 ± 0.02 |
| Likelihood of taking refuge (0/1) | 0.18 ± 0.05 | 0.18 ± 0.05 |
| Distance moved (cm) | 30.85 ± 6.26 | 35.25 ± 4.92 |
| Change in elevation (cm) | -3.85 ± 1.24 | -5.02 ± 1.06 |

**S3. Candidate model set from MARK model comparison.**

| **Model** | **Parameters** | **AIC** | **ΔAIC** |
| --- | --- | --- | --- |
| phi(.) p(maternal treatment) | 3 | 1442.93 | 0 |
| phi(.) p(maternal/enclosure treatment) | 5 | 1444.48 | 1.55 |
| phi(maternal treatment) p(maternal treatment) | 4 | 1444.56 | 1.63 |
| phi(maternal treatment + svl) p(maternal treatment) | 5 | 1446.56 | 3.63 |
| phi(maternal treatment + mass) p(maternal treatment) | 5 | 1446.59 | 3.66 |
| phi(maternal/enclosure treatment) p(maternal/enclosure treatment) | 8 | 1447.57 | 4.64 |
| phi(maternal treatment + mass + svl) p(maternal treatment) | 6 | 1448.55 | 5.62 |
| phi(maternal/enclosure treatment + mass) p(maternal/enclosure treatment) | 9 | 1449.57 | 6.64 |
| phi(maternal/enclosure treatment + svl) p(maternal/enclosure treatment) | 9 | 1449.63 | 6.7 |
| phi(maternal/enclosure treatment + mass + svl) p(maternal/enclosure treatment) | 10 | 1451.37 | 8.44 |
| phi(.) p(.) | 2 | 1453.88 | 10.95 |
| phi(.) p(enclosure type) | 3 | 1454.18 | 11.25 |
| phi(enclosure type) p(enclosure type) | 4 | 1454.94 | 12.01 |
| phi(enclosure type) p(.) | 3 | 1454.96 | 12.03 |
| phi(maternal treatment) p(.) | 3 | 1455.86 | 12.93 |
| phi(enclosure type + svl) p(enclosure type) | 5 | 1456.93 | 14 |
| phi(enclosure type + mass) p(enclosure type) | 5 | 1456.97 | 14.04 |
| phi(maternal/enclosure treatment) p(.) | 5 | 1457.39 | 14.46 |
| phi(enclosure type + mass + svl) p(enclosure type) | 6 | 1458.94 | 16.01 |
